# The Depreciation of Institutional Investments

**Published:** 1917-02-03

**Authors:** 


### The Leicester Royal Infirmary Meeting.

### THE DEPRECIATION OF INSTITUTIONAL INVESTMENTS.

THE freedom with which money quickly passes from hand to hand in the manufacturing centres, and the passing prosperity of industrial towns due to the war, is exemplified in the annual financial report of the Leicester Royal Infirmary submitted to the governors on Saturday, under the presidency of Mr. T. Fielding Johnson, jun., chairman of the Board.

The total net ordinary income for the year at £32,880 exceeded the expenditure by about £700. This was largely due to an effort by Mr. Alfred Corah, the institution's treasurer, and the High Sheriff of Leicestershire, who brought in an addition of £4,100 to the annual subscriptions list, and £350 to the donations list. These sums were, however, not all available as income, as it was the treasurer's definite object to set aside a sum of three or four thousand pounds each year to form a reserve fund for building and improvement purposes, with the object of avoiding intermittent appeals for money for extensions. Legacies to the general fund had produced £11,300, £3,000 of which had been applied in liquidating the balance brought forward on account of last year's investments, and the balance invested in War Loan. The invested capital of the institution had thus been increased by the entire legacies received during the year. The governors' decision to invest in the new War Loan should certainly appreciate the return on the institution's investments.

The feature of the year's work apart from the financial side is that, with a diminished number of beds available for civil needs, the patients were only slightly less than in the previous year, while the demands on the military wards averaged only eighty-five beds occupied during the year. There were no waiting patients on the civil list.

Progress has been made in the pathological department by the appointment of a permanent salaried pathologist, and so soon as the time is propitious it is hoped to proceed immediately with the building of the new pathological laboratories, *post-mortem* rooms, etc., for which the money has been raised. In connection with the treatment of venereal diseases, under the Local Government Board scheme, provision is being made for about six or seven beds, and for the appointment of male and female medical officers to undertake the work.

